# Supplementary material for: Gray matter networks associated with attention and working memory deficit in ADHD across adolescence and adulthood
Source: Transl Psychiatry. 2021 Mar 25;11:184. doi: 10.1038/s41398-021-01301-1 (PMC7994833; doi:10.1038/s41398-021-01301-1)
Supplement: Supplementary file 1 — Supplementary Materials [file 41398_2021_1301_MOESM1_ESM.pdf]

Supplementary text

Summary of analyses and results from the adult cohort reported in the previous paper by Duan et al. (1).

1. Participants include 301 samples from the NeuroIMAGE project (age range: [18, 30], female/male:127/174) and 185 samples from the Dutch chapter of the IMpACT consortium (age range: [18, 63], female/male:136/49). Adult ADHD patients met the DSM-IV or DSM-IV-TR criteria for adult ADHD, and had a formal or research diagnosis in childhood. For the NeuroIMAGE cohort, a total of 206 families were involved, including 124 independent participants, 71 two-member families, 11 three-or-four-member families. The participants from the IMpACT cohort were all independent. A total of 214 ADHD patients, 96 unaffected siblings and 176 healthy controls were studied. Two symptom domains, inattention (IA) and hyperactivity/impulsivity (HI), were evaluated for all participants based on 18 DSM-IV symptom questions. For working memory, the WAIS Digit Span task was employed for almost all participants (N=480) in both projects. For inhibition, the NeuroIMAGE project employed a stop task for 202 participants (Logan et al., 1984), while the IMpACT project utilized a go/no-go task for 150 subjects (Mostert et al., 2015b). Significant findings were only related to working memory.

2. Neuroimaging: T1-weighted MRI images were acquired with three 1.5T scanners with closely matched settings, such as, a voxel size of $1\times1\times1 mm^3$, TI 1000 ms, TR 2730 ms, TE 2.95 ms). After quality control all included sMRI scans were segmented into six types of tissues using Statistical Parametric Mapping 12. GM data were normalized to the MNI template, modulated and smoothed with a $6\times6\times6\ mm^3$ Gaussian kernel. A GM mask was applied to include voxels with mean gray matter density larger than 0.2. For each included voxel, the confounding effects of age, sex and site were corrected using a linear regression model, where the regression coefficients were estimated from data on controls only. After correcting age, sex and site effects, the total GM volume did not differ between the two cohorts ($p = 0.71$), and adding the cohort as an additional variable into the analyses did not change the significance of the reported results.

3. GM component decomposition and association analyses: GM data was decomposed into 22 distinct components using ICA implemented by the GIFT toolbox (http://mialab.mrn.org/software/gift). ICA decomposes the GM data into linear combinations of independent components; i.e., GM data (X) = loadings (A) × components (S). For extracted GM

components S (dimension: component ×voxel), each row is an independent component and represents the contributions of individual voxels to this component. For the loading matrix A (dimension: subject × component), each column is the loading vector of the corresponding component presenting the weights of this component across all subjects.

Associations between the GM components and the two symptom domains, as well as working memory and inhibition performance were tested using a linear mixed effect model, where family ID was used as a random effect to control for relatedness, and other predictors (i.e. age, sex and GM loading of a component) were treated as fixed effects. Additionally, within only patients and controls, GM components were tested for ADHD differences by similar linear mixed models controlling family ID. Significance was corrected for multiple comparisons for all components at a false discovery rate (FDR) of 0.05. Potential confounding effects of medication, comorbidity and IQ as well as cohort were also tested for these components by adding them as a covariate into the model separately. Moreover, pairwise group difference among medicated cases, medication-naïve cases, healthy controls and unaffected siblings were evaluated for GM identified components

4. VBM analysis: To identify GM regions underlying cognition or symptom, linear mixed effect models for GM density of each voxel were applied (cognition/symptoms = age + sex + site + GM of each voxel +family ID). Similarly, ADHD differences were tested on voxels of healthy controls and ADHD patients. Multiple comparison correction FDR $p < 0.05$ was applied, and a minimal of 10 voxels was set to include almost all identified regions.

5. ICA results: Out of 22 GM components extracted by ICA, four GM components were significantly related to either working memory or inattention symptom severity measures. GM loadings of component 1 in inferior semilunar lobule were positively related to forward digit span performance. GM loadings of component 2 in inferior frontal gyrus were positively associated with both forward and backward digit span performance. GM loadings of component 3 in superior and middle frontal gyri were positively related to backward digit span performance. GM loadings of component 4 encompassing cerebellar tonsil, culmen and tuber were negatively associated with inattention symptom severity.

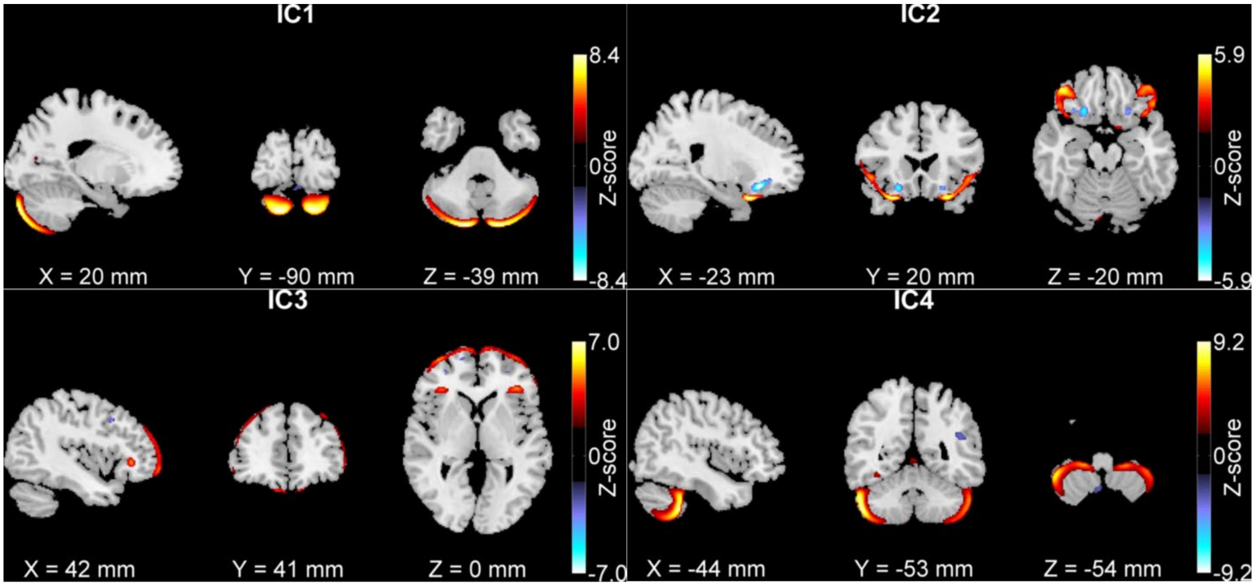

Figure SI. Four significant GM components (ICs) associated with either working memory or inattention symptoms (|Z| > 2.5). Figure comes from the paper by **Duan et al. (2018).**

In the case-control comparison, component 5, located in the middle frontal gyri in **Figure SII,** was identified showing significant GM anomalies. A significant medication effect was observed for component 5, and after regressing out the medication effect, the GM loadings still showed significant reduction in patients.

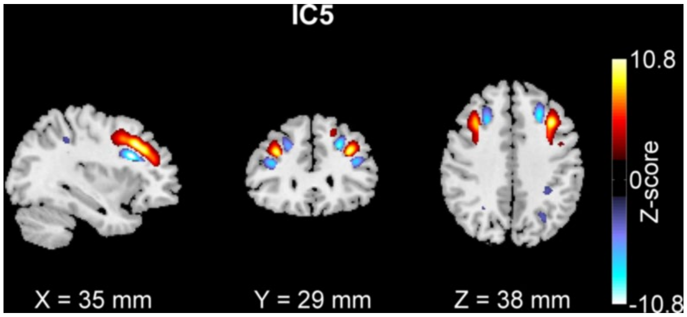

Figure II. One additional GM component (component 5 in middle frontal region) showing significant case-control. Figure comes from the paper by **Duan et al. (2018).**

VBM results: Multiple regions in frontal lobe, temporal lobe, and cerebellum in subplot A Figure SIII were significantly positively correlated with forward digit span performance. Regions in frontal lobe in subplot B Figure SIII were significantly and positively associated with backward digit span performance. Subplot C Figure SIII presents regions in cingulate gyrus showing a

significant GM reduction in ADHD patients. No voxels were significantly associated with inhibition or symptom domains.

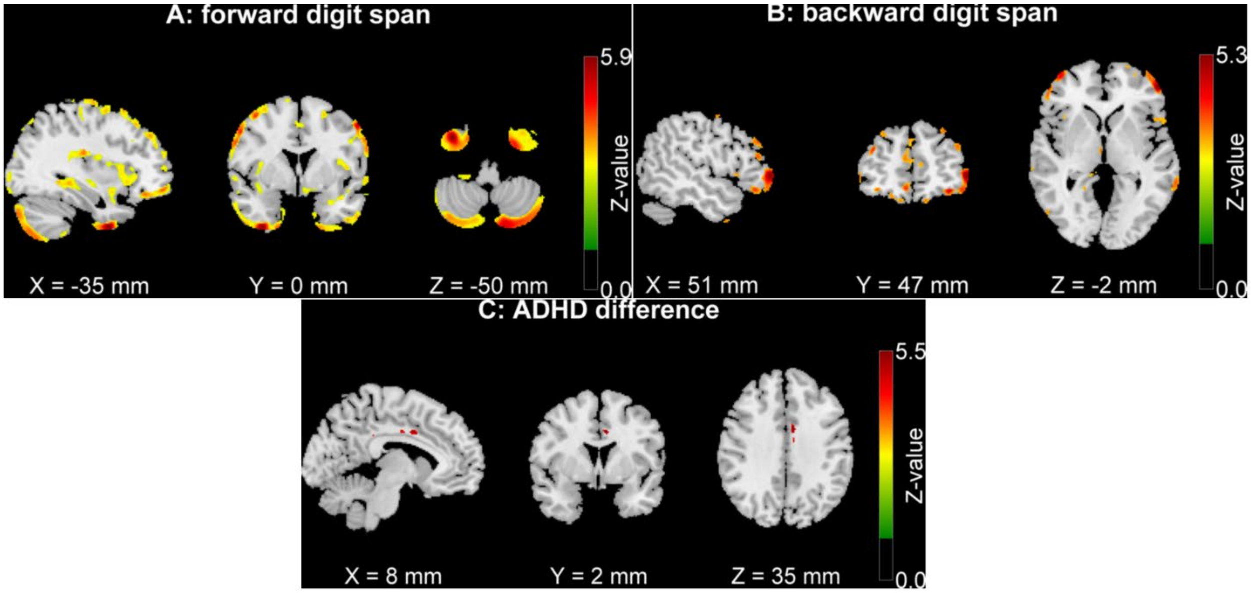

Figure SIII. Results of VBM analysis. Colors are coded for association coefficients, normalized Z scores. Figure comes from the paper by **Duan et al. (2018).**

Reference:

1. Duan K, Chen J, Calhoun VD, Lin D, Jiang W, Franke B, et al. (2018): Neural correlates of cognitive function and symptoms in attention-deficit/hyperactivity disorder in adults. *Neuroimage Clin*. 19:374-383.

Supplementary Table s1: Correlation between GM components derived from adults and adolescents

Adult IC	Correlation value *	Adolescent IC
# 1	0.68	# 7
# 2 (IC 1)	0.73	# 1
# 3	0.35	# 19
# 4	0.75	# 2
# 5	0.61	# 8
# 6 (IC 3)	0.26	# 14
# 7 (IC 5)	0.48	# 17
# 8	0.50	# 17
# 9	0.30	# 8
# 10	0.57	# 5
# 11	0.55	# 14
# 12 (IC 4)	0.49	# 13 **(IC A)**
# 13	0.33	# 6
# 14 (IC 6)	0.58	# 3 (IC B)
# 15	0.64	# 16
# 16	0.48	# 4
# 17 (IC2)	0.26	# 12
# 18	0.45	# 15
# 19	0.53	# 9
# 20	0.32	# 10
# 21	0.36	# 10
# 22	0.50	# 11

- All correlations with p<10E-16

Supplementary Table s2: Association results of the eight GM components using subsamples with limited age ranges. 427 adults with age between 18 and 40 years; 436 adolescents with age between 12 and 17 years old.

Adults components		Adults (age:18-40)	Adolescents (age: 12-18)
IC-1	case vs. control	*n.s*	$p = 2.07×10^{-2}$, reduction
	association	with working memory (forward), $p = 6.16×10^{-4}$, positive, $R^2 = 2.88\%$	with working memory (forward) *n.s.*
IC-2	case vs. control	*n.s.*	$p = 1.93×10^{-5}$, reduction
	association	with working memory (backward), $p = 1.56×10^{-3}$, positive, $R^2 = 2.32\%$	**with working memory (forward), $p = 9.12×10^{-2}$, positive, $R^2 = 0.65\%$;**
IC-3	case vs. control	*n.s.*	$p = 3.11×10^{-3}$, Reduction
	association	with working memory (backward), $p = 1.01×10^{-3}$, positive, $R^2 = 2.50\%$	with working memory (forward), $p = 5.70×10^{-2}$, positive, $R^2 = 0.839\%$
IC-4	case vs. control	$p = 4.11×10^{-2}$, reduction	$p = 8.45×10^{-3}$, reduction
	association	Inattentive symptom, $p = 1.18×10^{-2}$, negative, $R^2 = 1.47\%$; *	Inattentive symptom, $p = 2.56×10^{-2}$, negative, $R^2 = 0.71\%$
IC-5	case vs. control	$p = 4.70×10^{-5}$, reduction	*n.s.*
	association	*n.s.*	*n.s.*
IC-6	case vs. control	*n.s.*	$p = 9.51×10^{-3}$, reduction
	association	*n.s.*	with working memory (forward), $p = 3.00×10^{-2}$, positive, $R^2 = 1.07\%$
Adolescent components		Adults (age: 18-40)	Adolescents (age: 12-18)
IC-A	case vs. control	*n.s.*	$p = 6.15×10^{-4}$, reduction
	association	**with inattentive symptom, $p = 0.23$, negative, $R^2 = 0.25\%$;**	with inattentive symptom, $p = 3.54×10^{-3}$, negative, $R^2 = 1.17\%$
IC-B	case vs. control	*n.s.*	$p = 2.33×10^{-3}$, reduction
	association	with working memory (forward), $p = 2.78×10^{-2}$, positive, $R^2 = 1.17\%$. with working memory (backward), $p = 2.53×10^{-3}$, positive, $R^2 = 2.16\%$	with working memory (forward), $p = 2.24×10^{-2}$, positive, $R^2 = 1.17\%$; *

Results in bold failed passing p<0.05, while in full samples they were significant. Even though these associations failed replication of significance, but their directions were consistent with those in full samples.

* indicates these two associations were passing FDR correction in full samples, but they were now between p<0.05 and FDR corrected significance. Reduced sample size may partially contributed to this differences.

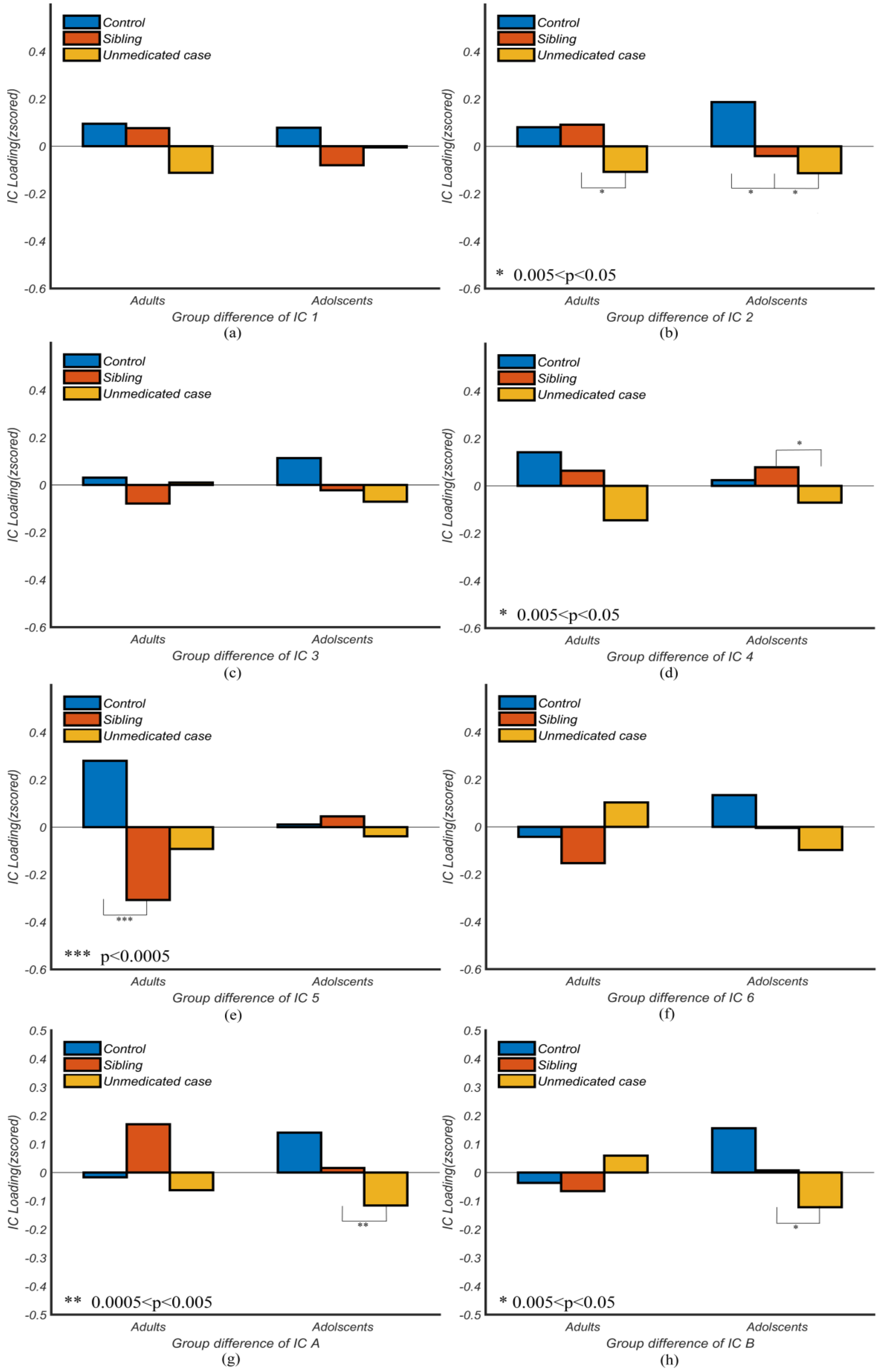

Control
Sibling
Unmedicated case
IC Loading(zscored)
0.4
0.2
0
-0.2
-0.4
-0.6
Adults
Adolscents
Group difference of IC 1
(a)
Control
Sibling
Unmedicated case
IC Loading(zscored)
* 0.005<p<0.05
Adults
Adolscents
Group difference of IC 2
(b)
Control
Sibling
Unmedicated case
IC Loading(zscored)
Adults
Adolscents
Group difference of IC 3
(c)
Control
Sibling
Unmedicated case
IC Loading(zscored)
* 0.005<p<0.05
Adults
Adolscents
Group difference of IC 4
(d)
Control
Sibling
Unmedicated case
IC Loading(zscored)
*** p<0.0005
Adults
Adolscents
Group difference of IC 5
(e)
Control
Sibling
Unmedicated case
IC Loading(zscored)
Adults
Adolscents
Group difference of IC 6
(f)
Control
Sibling
Unmedicated case
IC Loading(zscored)
0.5
0.4
0.3
0.2
0.1
0
-0.1
-0.2
-0.3
-0.4
-0.5
** 0.0005<p<0.005
Adults
Adolscents
Group difference of IC A
(g)
Control
Sibling
Unmedicated case
IC Loading(zscored)
* 0.005<p<0.05
Adults
Adolscents
Group difference of IC B
(h)

Figure S1. Comparison of GM volumes in unaffected siblings with controls and unmedicated patients. Nominal significant results ($p<0.05$) were indicated in the figure by asterisks. For adolescents, siblings showed mean GM volumes between unmedicated cases and controls in five components, and in three of those components' siblings showed at least nominal significant increase in GM volume than unmedicated cases. In adults,

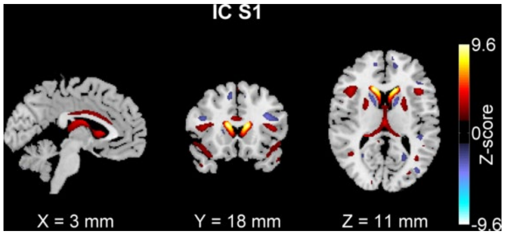

Figure S2. The caudate nucleus component in the adolescent group without voxel-wise age regression. This caudate component showed no difference between patients and controls, and no association with working memory or inattention.
